# Genomic Deletion of PFKFB3 Decreases In Vivo Tumorigenesis

**DOI:** 10.3390/cancers16132330

**Published:** 2024-06-26

**Authors:** Yoannis Imbert-Fernandez, Simone M. Chang, Lilibeth Lanceta, Nicole M. Sanders, Jason Chesney, Brian F. Clem, Sucheta Telang

**Affiliations:** 1 Department of Medicine, Division of Medical Oncology, Brown Cancer Center, University of Louisville, Louisville, KY 40202, USA; yoannis.imbertfernandez@louisville.edu (Y.I.-F.);; 2 Department of Pediatrics, University of Louisville, Louisville, KY 40202, USA; 3 Department of Biochemistry and Molecular Genetics, University of Louisville, Louisville, KY 40202, USA

**Keywords:** lung cancer, breast cancer, *Erbb2*, *Ras*, metabolism, phosphofructokinase, glycolysis, glucose metabolism

## Abstract

**Simple Summary:**

The breakdown of glucose in the glycolytic pathway provides energy and essential building blocks to support cell division and growth in important body processes such as embryo development but also can promote disease pathologies including tumor formation. A family of enzymes called the PFKFBs are important regulators of this pathway, and the PFKFB3 family member plays a key role. Examining the functions of PFKFB3 in live animals is critical for fully understanding its role in glycolysis, and we have developed a new mouse model that can knock out PFKFB3 to evaluate these functions. In our studies, we have found that these mice have decreased PFKFB3 levels and activity and that knocking out PFKFB3 markedly impairs the growth of tumors that closely mimic human cancer. Our data establish a new and effective model for examining the regulation of glucose metabolism by PFKFB3 and highlight its importance in tumor growth.

**Abstract:**

Rapidly proliferative processes in mammalian tissues including tumorigenesis and embryogenesis rely on the glycolytic pathway for energy and biosynthetic precursors. The enzyme 6-phosphofructo-2-kinase/fructose-2,6-bisphosphatase-3 (PFKFB3) plays an important regulatory role in glycolysis by activating the key rate-limiting glycolytic enzyme, 6-phosphofructo-1-kinase (PFK-1). We have previously determined that decreased PFKFB3 expression reduced glycolysis and growth in transformed cells in vitro and suppressed xenograft growth in vivo. In earlier studies, we created a constitutive knockout mouse to interrogate the function of PFKFB3 in vivo but failed to generate homozygous offspring due to the requirement for PFKFB3 for embryogenesis. We have now developed a novel transgenic mouse model that exhibits inducible homozygous pan-tissue *Pfkfb3* gene deletion (*Pfkfb3^fl/fl^*). We have induced *Pfkfb3* genomic deletion in these mice and found that it effectively decreased PFKFB3 expression and activity. To evaluate the functional consequences of *Pfkfb3* deletion in vivo, we crossed Cre-bearing *Pfkfb3^fl/fl^* mice with oncogene-driven tumor models and found that *Pfkfb3* deletion markedly decreased their glucose uptake and growth. In summary, our studies reveal a critical regulatory function for PFKFB3 in glycolysis and tumorigenesis in vivo and characterize an effective and powerful model for further investigation of its role in multiple biological processes.

## 1. Introduction

Glucose metabolism is an essential source of the biosynthetic intermediates and ATP required to support cell division and growth [1]. Rapidly proliferative processes in mammalian tissues including embryogenesis and tumor formation rely on glycolysis to fulfill their bioenergetic needs [1]. The first committed step in the glycolytic pathway is the conversion of fructose-6-phosphate (F6P) to fructose-1,6-bisphosphate by the enzyme, 6-phosphofructo-1-kinase (PFK-1), which therefore serves a critical and rate-limiting function in this pathway [2,3]. PFK-1 activity is tightly regulated by several effectors, among which fructose-2,6 bisphosphate (F26BP) has been found to be the most potent activator due to its function in increasing the affinity of PFK-1 for F6P and allosterically releasing the PFK-1 inhibition caused by ATP [4,5,6].

The steady-state intracellular concentrations of F26BP are controlled by a family of four bifunctional enzymes, termed the 6-phosphofructo-2-kinase/fructose-2,6-bisphosphatases (PFKFB1-4) [7]. These four enzymes (encoded by four separate genes, PFKFB1-4) phosphorylate F6P to F26BP and dephosphorylate F26BP to F6P and therefore play an important role in regulating glucose metabolism. The PFKFB isoforms show high sequence homology in their core catalytic domains but display variations in their kinase and bisphosphatase activities which drive their relative contributions to intracellular F26BP levels [7,8]. Of the PFKFBs, the PFKFB3 enzyme has been determined to have the highest kinase activity (with a kinase/bisphosphatase ratio of 740:1) [7,9]. Although co-expression of several isoforms that contribute to F26BP production has been demonstrated in mammalian tissues [10], the high kinase activity exhibited by PFKFB3 suggests that this enzyme may contribute significantly to the intracellular F26BP concentration and drive tissue glycolysis. 

Several factors and stimuli, including hypoxia-inducible factor-1α [11,12], estrogen [13], progestins [14], growth factors [15,16], and proinflammatory molecules [17], have been demonstrated to actively promote the expression of the PFKFB3 enzyme and strongly indicate a prominent role for this enzyme in regulating glycolysis and proliferation in diverse processes, including inflammation, host defense, and cancer. Of these functions, the role of PFKFB3 in tumor glucose metabolism has been extensively interrogated. Increased PFKFB3 expression has been demonstrated in multiple tumor types [7]. In addition to the stimuli noted above, PFKFB3 expression and kinase activity are demonstrated to be increased through several mechanisms including the loss of the tumor suppressor phosphatase and tensin homolog (PTEN), which both decreases PFKFB3 degradation by Anaphase Promoting Complex (APC)/cdc20 homolog 1(Cdh1) [18] and allows the activation of protein kinase B (PKB, Akt) which then activates PFKFB3 by phosphorylation [19] and through phosphorylation driven by other mechanisms [20]. In addition to the role played by PFKFB3-driven F26BP in regulating glycolysis, it has also been found to activate cyclin-dependent kinases to increase proliferation [21]. Given the key function of F26BP in tumor glycolysis and growth, the high kinase activity of PFKFB3 and its production of F26BP indicate an important role for this enzyme in tumor proliferation and glucose metabolism.

Previous studies from our laboratory have determined that decreased PFKFB3 expression and activity reduced glycolysis and suppressed proliferation in transformed cells [10]. Interestingly, our manipulations of PFKFB3 showed that decreasing its expression led to substantially greater suppression of anchorage-independent growth of these cells as a three-dimensional mass, indicating that PFKFB3-regulated glycolytic activity may support similar processes such as tumor growth and embryogenesis [10]. A critical role for PFKFB3-driven glycolysis in regulating tumor growth was confirmed by our examination of a constitutive knockout mouse model of the *Pfkfb3* gene where we found that PFKFB3 was required for oncogene-driven anchorage-independent growth in vitro and xenograft growth in vivo [10,22]. These studies also revealed that PFKFB3 was essential for embryo development, and due to this requirement, we were unable to generate homozygous knockout mice to study the role of this enzyme in adult animals [22].

A growing body of data has implicated the glycolytic pathway in supporting multiple physiological and pathological processes, including immune cell development and function, neural and organ development, angiogenesis, tumorigenesis, and chemoresistance [23,24,25,26,27,28,29]. As a glycolytic regulator, PFKFB3 performs a critical function in these processes, and accordingly, the development of a PFKFB3 homozygous knockout model is vital for the accurate assessment of its role. The recent development of novel small molecule inhibitors of PFKFB3 for therapeutic use in cancer has added a more immediate and significant relevance to these studies [30,31]. Global genomic *Pfkfb3* deletion will mimic the systemic effects of PFKFB3 inhibition, and an evaluation of its effects will serve to inform the further development of PFKFB3 inhibitors for clinical use in patients. 

To interrogate the glycolytic function of the PFKFB3 enzyme in adult animals, we have generated a novel transgenic mouse model that allows inducible homozygous pan-tissue *Pfkfb3* gene disruption. Based on prior data, we postulate that genomic deletion of *Pfkfb3* will restrict rapidly proliferative processes such as tumorigenesis but permit the proliferation and growth of untransformed normal organs and tissues in adult mice [22]. In these studies, we have demonstrated effective Cre-mediated recombination in our inducible *Pfkfb3* knockout and the efficacy of genomic deletion of *Pfkfb3* in decreasing PFKFB3 protein expression and activity. We also have evaluated the functional effects of *Pfkfb3* deletion in relevant oncogene-driven spontaneous models of cancer where we have found that PFKFB3 enzyme activity is required for tumor initiation and growth. Our data confirm a key function for PFKFB3 in regulating glycolysis in tumorigenesis in vivo and additionally provide rationale for the further development of PFKFB3 inhibitors as cancer therapeutics.

## 2. Materials and Methods

### 2.1. Generation of Conditional Pfkfb3 Knockout Mice

Our strategy for generating *Pfkfb3* knockout mice targeted exons 2 and 3 of the wild-type (WT) mouse *Pfkfb3* gene. Mice were generated in collaboration with InGenious Targeting Laboratory (iTL, Ronkonkoma, NY, USA). The targeting vector contained LoxP sites recognized by Cre recombinase and FRT sites recognized by Flp recombinase. For the construction of the targeting vector, an 11.5 kb region was sub-cloned from a positively identified C57BL/6 (RPCI23) BAC clone. The region was designed such that the short homology arm (SA) extended 1.57 kb 3′ to exon 3, and the long homology arm (LA) ended 5′ to exon 2 and was ~7.4 kb long. The loxP-flanked Neomycin resistance cassette was inserted on the 3′ side of exon 3, and a single loxP site was inserted at the 5′ side of exon 2. The size of the conditionally targeted region was ~2.55 kb and included exons 2–3. The targeting vector was confirmed by restriction analysis after each modification step, linearized, and transfected by electroporation of C57BL/6 × 129/SvEv hybrid embryonic stem (ES) cells. After selection in antibiotic (G418), surviving clones were expanded for PCR analysis to identify recombinant ES clones. High-percentage chimeras were obtained by ES cell blastocyst injection. The introduction of Flp recombinase, achieved by crossing chimeras with C57Bl/6 FLP mice (obtained from Jackson Laboratory, Bar Harbor, ME, USA, stock #003800), removed the Neo cassette, leaving exons 2 and 3 flanked by LoxP sites to generate *Pfkfb3*^fl/fl^ mice. We then generated Cre/*Pfkfb3*^fl/fl^ mice by crossing *Pfkfb3*^fl/fl^ mice with CAGGCre-ER mice (Jackson Lab, stock #004682) [32]. The CAGGCre-ER mice contain a tamoxifen (TAM)-inducible Cre-recombination system driven by the chicken beta-actin promoter/enhancer and therefore cause deletion of floxed sequences in multiple tissues [32].

### 2.2. Fibroblast Isolation and In Vitro Cre-Mediated Recombination

Ear pinna fibroblasts were isolated from untreated Cre/*Pfkfb3*^fl/fl^ mice. Pinna tissue was cut into 2–3 mm fragments and incubated with trypsin-EDTA (0.25%, Invitrogen, Grand Island, NY, USA) at 37 °C for 1 h and then centrifuged. Trypsin was then removed, and tissue fragments were resuspended in Dulbecco’s modified Eagle medium (DMEM) containing fetal calf serum (FCS) (both from Invitrogen) and incubated in tissue culture plates at 37 °C in 5% CO_2_ to allow attachment. For experiments, fibroblasts were plated in 6-well plates and treated with ethanol ± 4-hydroxytamoxifen (4OHT, Sigma-Aldrich, St. Louis, MO, USA) at indicated concentrations for 72 h prior to harvest and examination for PFKFB3 expression and activity.

### 2.3. Western Blotting

Cells were harvested, washed X1 in PBS, and lysed in 1X lysis buffer (Pierce Biotechnology, Rockford, IL, USA) containing protease inhibitors (1:100, Protease Inhibitor Cocktail Set V, Sigma, St. Louis, MO, USA). Protein samples were resolved on 4–20% SDS-PAGE gels (BioRad, Hercules, CA, USA) and transferred to PVDF membranes (BioRad). After blocking in Tris-buffered saline containing 0.1% Tween 20 (TBS-T, Boston Bioproducts, Milford, MA, USA) and 5% milk, membranes were probed with antibodies to PFKFB3 (1:1000 in 2.5% milk in TBS-T overnight at 4 °C, Proteintech, Chicago, IL, USA) or β-actin (1:5000 in 2.5% milk in TBS-T for 1 h, room temperature, Sigma). Secondary antibodies used were HRP-conjugated goat anti-rabbit (for PFKFB3) or anti-mouse (for β-actin), both at 1:7500 dilution in TBS-T (both antibodies from Pierce Biotechnology). ECL Prime Western blotting detection reagent (Cytiva, Amersham, Little Chalfont, Buckinghamshire, UK) was used to detect antibody binding. Scanned images were quantified by densitometric analyses using Image J software (http://rsb.info.nih.gov/ij/, 12 November 2023). Values obtained were normalized to β-actin and expressed in densitometric units as a percentage of control. The data presented are the mean ± SD from triplicate measurements from three independent experiments. Statistical significance was assessed by the two-sample *t* test (independent variable).

### 2.4. F26BP Measurements 

Cells were lifted using trypsin, washed once with phosphate-buffered saline (PBS, Invitrogen), and then stored as pellets at −80 °C until use. Tissues and organs were snap-frozen in liquid nitrogen at harvest and stored at −80 °C until use. For use, frozen tissue was pulverized using a Bessman pulverizer (Spectrum Laboratories, New Brunswick, NJ, USA) following the manufacturer’s instructions. NaOH and Tris acetate were added to cell pellets and tissues which were then lysed by heating at 80 °C for 5 min. Lysates were neutralized to a pH of 7.2 using cold HEPES and acetic acid. F26BP content was measured as previously described, and its concentration was normalized to total cellular protein using the bicinchoninic acid assay (BCA assay, Thermofisher, Rockford, IL, USA) [33]. All data are expressed as the mean ± SD of three experiments. Statistical significance was assessed by the two-sample *t* test (independent variable).

### 2.5. Glycolysis Assay

Ear-derived fibroblasts treated with vehicle (ethanol) ± 4OHT were incubated in DMEM (500 μL) containing 1 μCi of 5-[^3^H] glucose (Cambridge Isotope Laboratories, Tewksbury, MA) for 1 h at 37 °C in 5% CO_2_. Media were then collected, ^3^H_2_O formed from 5-[^3^H] glucose was measured, and counts were normalized to protein concentration as previously described [34]. All data are expressed as the mean ± SD of three experiments. Statistical significance was assessed by the two-sample *t* test (independent variable).

### 2.6. Immunohistochemistry 

For immunohistochemistry, 5 μm sections of formalin-fixed and paraffin-embedded tissue mounted on slides were deparaffinized using xylene, and then epitope retrieval was conducted using citrate buffer in a 2100 Retriever (Electron Microscopy Sciences, Hatfield, PA, USA). Hematoxylin and eosin (H & E) staining was performed using standard procedures. For immunohistochemistry, tissue sections were first blocked with 10% goat serum for 60 min. Serial sections were next incubated with primary antibodies against PFKFB3 (Proteintech, Rosemont, IL, USA), cleaved caspase-3 (Cell Signaling, Danvers, MA, USA), Ki 67 (Abcam, Boston, MA, USA), or Cyclin D1 (Santa Cruz Biotechnology, Dallas, TX, USA) overnight, followed by HRP-linked goat anti-rabbit or anti-mouse secondary antibodies (1:300, Pierce Biotechnology, Rockford, IL, USA). Tissue sections were developed with 3,3′-diaminobenzidine tetrahydrochloride (DAB, Vector Laboratories, Burlingame, CA, USA); nuclei were counterstained (Mayer’s hematoxylin, Sigma-Aldrich), and coverslips attached (Permount, Fisher Scientific, Fairlawn, NJ, USA). Slides were scanned by a ScanScope XT Digital Slide Scanner (Aperio, Leica, Deerpark, IL, USA). Data were analyzed using the positive pixel count algorithm (ImageScope, Aperio) or by direct counting of positive cells in a minimum of 5 fields of 1 mm^2^ for each tumor section. The data are depicted as % positive pixels/total pixels ± SD or number of positive cells/mm^2^ ± SD. Statistical significance was assessed by the two-sample *t* test (independent variable). 

### 2.7. Tumor Model Generation

For the generation of a mammary tumor model, Cre/*Pfkfb3*^fl/fl^ mice were crossed with a spontaneous mammary tumor model (*Erbb2* mice, Jackson Lab, stock #005038 [35]) to generate *Erbb2*/Cre/*Pfkfb3*^fl/fl^ mice, and offspring were interbred to generate mice with *Erbb2* expression levels equivalent to homozygous expression levels to use for studies. To generate a lung tumor model, Cre/*Pfkfb3*^fl/fl^ mice were crossed with a spontaneous lung tumor model (*K-ras^LA1^* mice, NCI Mouse Repository, Frederick, MD, USA [36]) to generate *K-ras^LA1^*/Cre/*Pfkfb3^f^*^l/fl^ mice. Recombination was achieved by intraperitoneal injections of TAM (Sigma-Aldrich, St. Louis, MO, USA) dissolved in corn oil (Sigma-Aldrich) at a dose of 9 mg/40 g weight for 5 days as previously described [32]. Control mice received an intraperitoneal (IP) injection of corn oil (as vehicle) for the same period of time. Cre/*Pfkfb3*^fl/fl^ mice were analyzed for PFKFB3 protein expression 5 days after the completion of injections. Treatment was started in *Erbb2*/Cre/*Pfkfb3*^fl/fl^ mice with corn oil ± TAM when a measured tumor burden of ~100 mg was noted and in *K-ras^LA1^*/Cre/*Pfkfb3*^fl/fl^ mice at 6 weeks of age. 

All animal experiments were performed in compliance with practices described in the National Institutes of Health Guide for Care and Use of Laboratory Animals and were approved by the Institutional Animal Care and Use Committee at the University of Louisville. Mice (females only for the *Erbb2* model and males and females for the *K-ras^LA1^* model) were randomly allocated to treatment and control groups. Investigators were blinded to group allocation for the duration of the experiment. 

### 2.8. Tumor Growth Monitoring

Mammary tumors were followed from the time of appearance until endpoint (defined as the point when control tumors reached 10% body mass, when tumor ulceration was noted, or when animals showed signs of distress (including changes in activity or behavior, weight loss, or abnormal examination)), and lung-tumor-bearing mice were followed until clinical signs of distress (including tachypnea, changes in activity or behavior, weight loss, or abnormal examination) were noted in either group. Researchers who were blinded used microcalipers to determine mammary tumor burden according to the following formula: weight (mg) = (width, mm)^2^ × (length, mm)/2 [37]. At endpoint, groups of tumor-bearing mice (n = 4) were anesthetized with 2% isoflurane in oxygen and then scanned for 10 min in a micro-CT scanner (CTI Concorde Microsystems, Knoxville, TN, USA). To conduct positron emission tomographic (PET) imaging, tumor-bearing mice were injected intravenously with 2-[^18^F]-fluoro-2-deoxyglucose (FDG, 150µCi in water, 100 µL/mouse). After 45 min, mice were anesthetized (with 2% isoflurane/oxygen) and imaged in an R-4 Rodent micro-PET Scanner (CTI Concorde Microsystems). Regions of interest in tumors and the cerebellum were quantified in quadruplicate and expressed as the mean ± SD of the ratio of tumor/cerebellar FDG uptake. Animals were euthanized at the endpoint, and tumors and/or lungs were removed and fixed in 10% buffered formaldehyde and paraffin-embedded to stain for histopathology (H & E) and for immunohistochemistry as described above. Blood samples were taken by cardiac puncture for analyses at the University of Louisville Comparative Medicine Research Unit. All data are expressed as the mean ± SD of two experiments. Statistical significance was assessed by the two-sample *t* test (independent variable).

### 2.9. Statistics and Sampling

All data were analyzed using GraphPad Prism (Version 7.04, San Diego, CA, USA). All experiments compared means between two groups (control and treatment/deletion) with independent samples and equal variance for which significance was calculated using the Student’s *t* test. A *p* value of <0.05 was considered statistically significant.

In order to minimize bias and ensure randomization in our in vivo experiments, mice were randomly assigned to treatment or control groups with the assistance of an online tool from Graphpad Prism (www.graphpad.com/quickcalcs/randomize1/, accessed 14 July 2020). At endpoint, samples were taken from all the mice in control and treatment groups, and three replicates from each organ were examined in the described assays. Additionally, three replicates were examined from each sample for all in vitro assays. For immunohistochemistry, 5 random 1 mm^2^ areas were chosen from each section evaluated, and positive cells were counted or staining was evaluated using the positive pixel algorithm (Aperio Imagescope). To further reduce bias, researchers conducting assays and evaluating results and immunostaining were blinded to sample identifiers.

## 3. Results

### 3.1. Generation of a Transgenic Pfkfb3 Inducible Knockout Mouse

**Figure 1:**
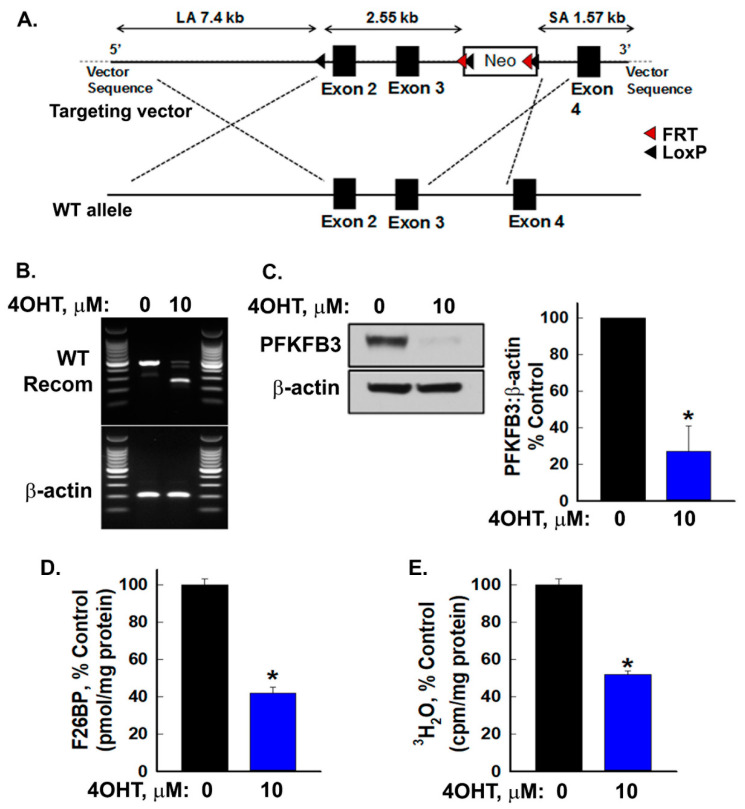
Generation of floxed *Pfkfb3* mice and confirmation of effective genomic deletion in vitro. Schematic showing the conditional targeting vector used for generation of floxed *Pfkfb3* mice (**A**). Ear fibroblasts isolated from tamoxifen (TAM)-inducible Cre/*Pfkfb3*^fl/fl^ mice were plated and exposed to vehicle (ethanol) or 10 µM 4-hydroxytamoxifen (4OHT) for 72 h and then harvested and analyzed for recombination (**B**); PFKFB3 protein expression and densitometry (**C**); fructose-2,6 bisphosphate (F26BP) levels (normalized to protein concentration) (**D**); and glycolysis (**E**). Data are expressed as the mean ± SD of three experiments, * *p* value < 0.05 relative to vehicle.

In order to fully evaluate the role of the PFKFB3 enzyme in vivo, we sought to create an inducible knockout mouse model that would allow the temporally regulated inactivation of the *Pfkfb3* gene. We generated a *Pfkfb3* floxed mouse wherein exons 2 and 3 of the *Pfkfb3* gene were flanked by LoxP sites (Figure 1A). We then crossed homozygous floxed *Pfkfb3*^fl/fl^ mice with mice expressing Cre recombinase under the control of a tamoxifen-inducible beta-actin promoter/enhancer which has been demonstrated to drive Cre recombinase expression in a wide spectrum of cell types and tissues [32].

As a precursor to the manipulation of gene activity in the whole organism, we initially examined the efficacy of recombination and deletion of *Pfkfb3* in vitro by using ear pinna fibroblasts isolated from Cre-bearing homozygous *Pfkfb3*^fl/fl^ mice. We expanded the fibroblasts in vitro and exposed them to vehicle ± 4OHT for 72 h. We first confirmed effective recombination and genomic deletion by conducting PCR on genomic DNA extracted from the cells and then lysed treated fibroblasts and confirmed decreased PFKFB3 protein expression (Figure 1B,C and Figure S1).

The PFKFB3 enzyme has previously been established to have a significantly higher kinase activity than its bisphosphatase activity, indicating its importance in modulating cellular F26BP levels [38]. Based on these data, we predicted that genomic deletion of *Pfkfb3* and the resultant decrease in PFKFB3 protein would decrease intracellular levels of F26BP and, as a result, decrease the rate of glycolysis. We found that 4OHT-driven recombination and *Pfkfb3* deletion did cause a marked decrease in cellular F26BP levels and glycolysis in vitro (Figure 1D,E).

### 3.2. Homozygous Pfkfb3 Deletion in Adult Mice Does Not Affect Organ Function In Vivo

**Figure 2:**
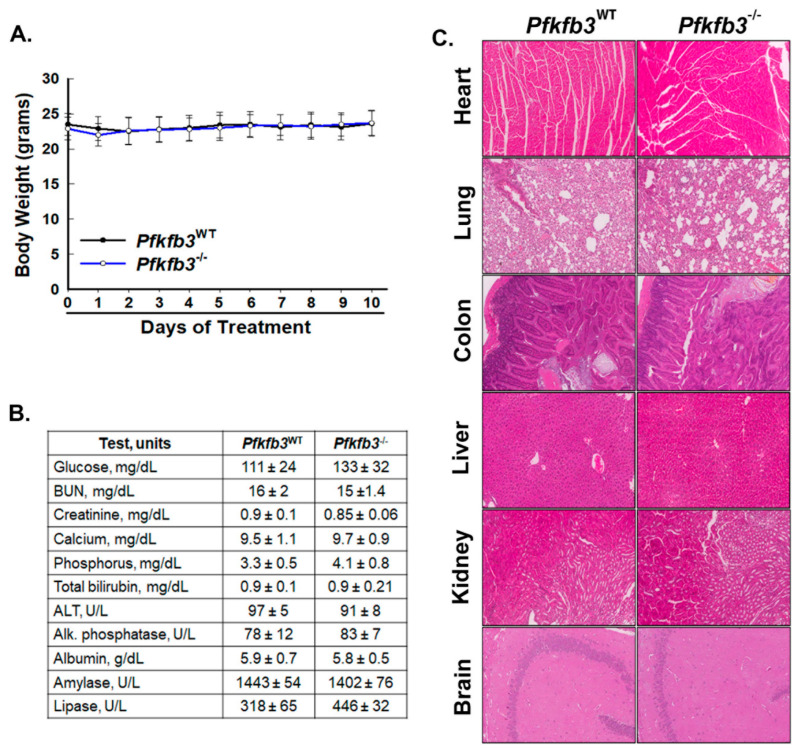
Homozygous genomic deletion of *Pfkfb3* causes no adverse effects on organ histology or function. Groups of Cre/*Pfkfb3*^fl/fl^ mice were administered corn oil as vehicle (*Pfkfb3*^WT^) ± TAM (*Pfkfb3^−/−^*) intraperitoneally for 5 days. Body weights were monitored during treatment and for 5 days following completion of treatment (**A**). Mice were euthanized 5 days after completion of treatment, and blood was sampled for tests as detailed (**B**). Organs were harvested and sections stained with hematoxylin & eosin (H & E) for examination (representative sections shown, 10× magnification) (**C**).

Our previous studies aiming to develop a mouse model of constitutive homozygous *Pfkfb3* deletion demonstrated that PFKFB3-driven glycolysis plays a critical role in supporting embryogenesis and organ development [22]. However, our inability to generate homozygous *Pfkfb3* knockout offspring precluded an assessment of the requirement for PFKFB3 for organ function in adult animals. Before embarking on an extensive examination of the effects of genomic deletion of *Pfkfb3* in adult homozygous mice, we first assessed the possibility of deleterious effects of decreased PFKFB3 expression on organ histology and function in vivo. We administered corn oil (as vehicle) or TAM in corn oil for 5 days intraperitoneally to 16-week-old TAM-inducible Cre-bearing homozygous *Pfkfb3*^fl/fl^ mice and followed the animals for 5 days after the completion of administration. We found no adverse effects of TAM administration or *Pfkfb3* genomic deletion on body mass (Figure 2A) or activity and did not observe any signs suggestive of distress. We euthanized groups of control and TAM-treated mice 5 days after the completion of treatment and confirmed recombination in organs and then examined serial sections of the indicated organs for histopathological changes (using H & E staining, Figure 2C). We also examined blood serum samples for signs suggestive of organ dysfunction and found that the animals tolerated *Pfkfb3* deletion without any noted evidence of organ pathology or dysfunction (Figure 2B,C). 

### 3.3. Cre-Mediated Recombination Effectively Decreases PFKFB3 Protein Expression and Activity in Diverse Organs In Vivo

**Figure 3:**
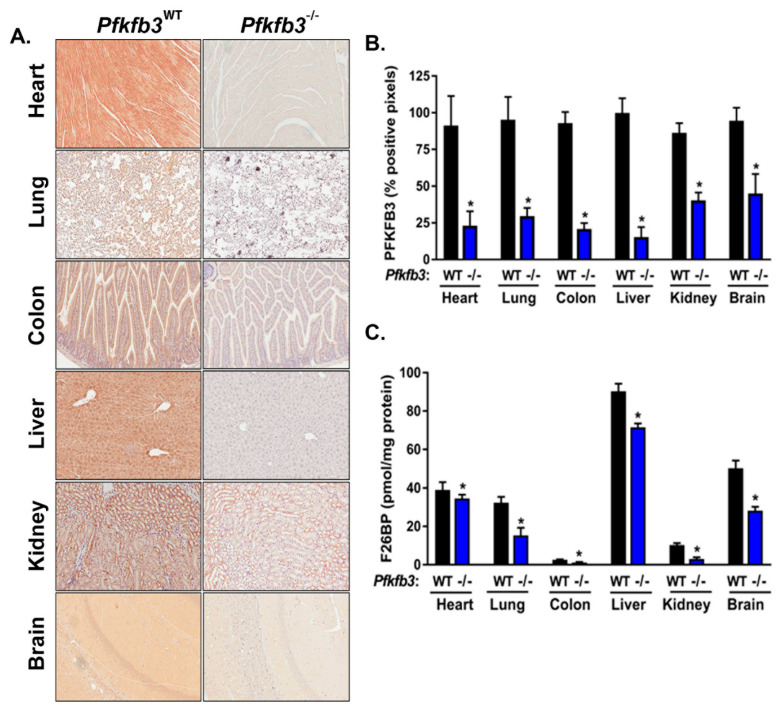
*Pfkfb3* gene deletion in adult mice effectively decreases PFKFB3 expression and activity in multiple organs. Groups of Cre/*Pfkfb3*^fl/fl^ mice were administered corn oil (*Pfkfb3*^WT^) ± tamoxifen (*Pfkfb3^−/−^*) intraperitoneally for 5 days and euthanized after an additional 5 days. Organs were harvested and sections examined by immunohistochemistry for PFKFB3 expression (representative sections are shown, 10× magnification) (**A**), and PFKFB3-positive pixels were enumerated in a minimum of 5 fields per tissue section (**B**). Organs were analyzed for F26BP (normalized to protein concentration) (**C**). Data are expressed as the mean ± SD, * *p* values < 0.05 relative to vehicle controls.

We next tested the efficiency of Cre-mediated induction of recombination and genomic deletion in adult tissues in our model by the administration of intraperitoneal TAM in the groups of 16-week-old adult homozygous Cre/*Pfkfb3*^fl/fl^ mice treated with corn oil ± TAM noted above. We found that TAM administration for 5 days caused a significant decrease in PFKFB3 protein expression in all organs examined by immunohistochemical analysis and confirmed by densitometry (Figure 3A,B). We observed variations in PFKFB3 protein expression in different organs, which we surmise are due to variations in Cre expression and activation as has been previously described [32]. To evaluate the effects of decreased PFKFB3 protein expression on PFKFB3 enzyme activity, we measured F26BP concentrations in the examined organs. We found that F26BP levels were decreased in all organs examined in TAM-treated mice relative to vehicle-treated mice (Figure 3C). We also found variations in the effect of *Pfkfb3* deletion on F26BP levels in the examined organs which we anticipated based on the known co-expression of other PFKFB isoforms in various organs that contribute to intracellular steady-state F26BP levels [34]. 

### 3.4. Pfkfb3 Deletion Decreases Glucose Uptake and Growth of Spontaneous HER2-Driven Mammary Tumors

**Figure 4:**
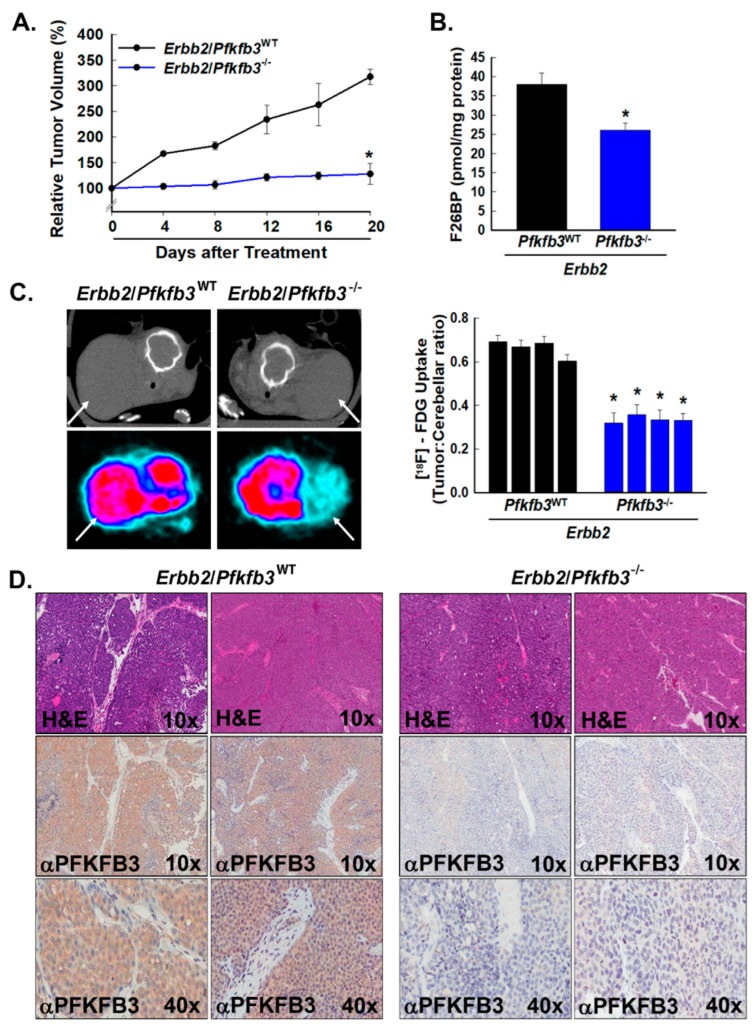
*Pfkfb3* genomic deletion decreases glucose uptake and growth in HER2-driven mammary tumors. Groups of *Erbb2*/Cre/*Pfkfb3*^fl/fl^ mice were followed from the appearance of mammary tumors until the tumors were ~100 mg then randomized (n = 6/group) to treatment with vehicle (*Erbb2*/*Pfkfb3*^WT^) ± TAM (*Erbb2*/*Pfkfb3*^−/−^). Tumor growth was followed with calipers every 4 days until the endpoint (data shown as relative increase in tumor volume from baseline tumor measurements) (**A**). At the endpoint, mice were euthanized, and mammary tumors were extracted and analyzed for F26BP (normalized to protein) (**B**). At the endpoint, after 2-[^18^F]-fluoro-2-deoxyglucose (FDG) administration, micro-CT (left, upper panels) and positron emission tomographic (PET) scans (left, lower panels) were obtained; representative transverse view cuts are shown with white arrows indicating tumors and regions of interest in the tumor and cerebellum quantified in quadruplicate (right) (**C**). Serial sections of tumors from *Erbb2*/*Pfkfb3*^WT^ and *Erbb2*/*Pfkfb3*^−/−^ mice were stained with H & E (representative sections at 10× magnification shown) and examined by immunohistochemistry for PFKFB3 protein expression (representative sections at 10× and 40× magnification shown) (**D**). Data are expressed as the mean ± SD, * *p* values < 0.05 relative to vehicle controls.

Tumors rely on a high rate of glucose metabolism to supply energy and biosynthetic precursors required to support their growth [1]. Given the previously described regulatory role played by PFKFB3-generated F26BP in glycolysis, we postulated that decreased PFKFB3 expression due to genomic deletion of *Pfkfb3* would significantly restrict tumor growth. We sought to evaluate the effect of decreased PFKFB3 expression in a tumor model that accurately recapitulates human disease and therefore employed a model of spontaneous oncogene-driven mammary tumor development that relies on the transgenic expression of activated *Erbb2* (HER2/neu) under the control of the mouse mammary tumor virus (*Erbb2* mice) [35]. To bolster the clinical relevance of our experiments, we examined the effect of *Pfkfb3* deletion on established mammary tumors to mimic conditions wherein therapeutic intervention by manipulation of PFKFB3 activity might be anticipated. To this end, we followed homozygous female *Erbb2*/Cre/*Pfkfb3*^fl/fl^ mice for the development of palpable mammary masses and, when tumor development was observed and burden reached ~100 mg, randomized mice to intraperitoneal administration of vehicle ± TAM for 5 days. We monitored the mammary tumor burden in the groups by caliper measurements and found that TAM-induced *Pfkfb3* deletion led to a marked suppression of tumor growth (Figure 4A). These results correlate with similar antitumor effects seen in *Erbb2* transgenic mice using a specific small molecule PFKFB3 inhibitor and additionally confirm the on-target effects of the inhibitor [39]. To control for unanticipated effects of TAM on tumor growth, we also examined tumor growth in *Erbb2*/Cre/*Pfkfb3*^+/+^ mice (WT for *Pfkfb3*) with and without TAM administration and observed similar growth in both groups (Figure S2). At the endpoint, we examined the tumors by micro-CT and measured tumor FDG uptake by PET in vehicle- and TAM-treated groups and found markedly reduced FDG uptake in the mammary tumors in TAM-treated mice relative to the vehicle-treated group (Figure 4C) as has also been described as a result of PFKFB3 inhibition and heterozygous genomic deletion of *Pfkfb3* [10,31]. We then resected the mammary tumors from vehicle- and TAM-treated mice, measured their intracellular F26BP concentration, and found a significant reduction in F26BP levels in the TAM-treated tumors relative to the control group (Figure 4B). We additionally confirmed markedly lower PFKFB3 expression in the TAM-treated tumors relative to control tumors by immunohistochemistry (representative sections shown, Figure 4D). 

### 3.5. Pfkfb3 Genomic Deletion Causes Apoptosis and Cell Cycle Arrest in Spontaneous HER2-Driven Mammary Tumors

**Figure 5:**
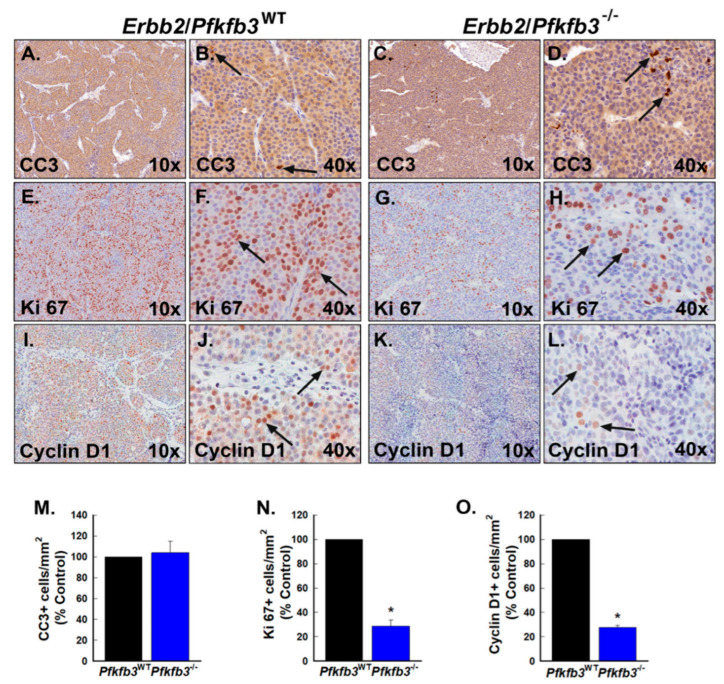
*Pfkfb3* gene deletion causes cell cycle arrest in HER2-driven mammary tumors. (**A**–**L**). Serial sections of tumors from *Erbb2*/*Pfkfb3*^WT^ and *Erbb2*/*Pfkfb3*^−/−^ mice were examined by immunohistochemistry for cleaved caspase 3 (CC3), Ki67, and Cyclin D1 expression (representative sections at 10× and 40× magnification shown). Representative positive cells are indicated by black arrows. CC3-, Ki67-, and Cyclin D1-positive cells were enumerated in 5 mm^2^ fields per tumor section from *Erbb2*/*Pfkfb3*^WT^ and *Erbb2*/*Pfkfb3*^−/−^ mice and are presented as indicated (**M**–**O**). Data are expressed as the mean ± SD, * *p* value < 0.05 relative to vehicle controls.

Previous data from our laboratory have shown that PFKFB3 inhibition reduced tumor cell viability and xenograft growth in part by inducing apoptosis [31]. We therefore speculated that reduced tumor growth caused by *Pfkfb3* deletion might be due in part to an increase in apoptosis. We examined sections from tumors harvested from vehicle- and TAM-treated *Erbb2*/Cre/*Pfkfb3*^fl/fl^ mice for the presence of apoptotic cells by immunohistochemistry for cleaved caspase 3 (CC3) but observed only a minimal increase in CC3-positive cells in these tumors relative to vehicle-treated control tumors (Figure 5A–D,M). Since our studies have previously determined that small molecule inhibition of PFKFB3 decreased cell proliferation and growth by an arrest of cell cycle progression at the G1 phase [21], we next examined the tumors for evidence of cell cycle arrest. The expression of Ki67 correlates with the later S and G2 phases of the cell cycle, and we therefore postulated that G1 arrest would result in reduced numbers of Ki67-positive cells in the TAM-treated tumors [40]. We examined tumor sections by immunohistochemistry for Ki67 expression and found a marked decrease in Ki67-positive cells in TAM-treated tumors relative to control tumors suggesting that genomic deletion of *Pfkfb3* decreases cell cycle progression in vivo (Figure 5E–H,N). 

The cyclin proteins are key regulators of cell cycle progression, and Cyclin D1, in particular, has been interrogated extensively for its role in modulating the progression from the G1 to the S phase of the cell cycle by regulating CDK4 and CDK6 [41]. Based on our Ki67 data suggesting that decreased PFKFB3 expression may cause a G1 arrest, we also examined Cyclin D1 expression in vehicle- and TAM-treated tumors and found a profound decrease in Cyclin D1 expression in TAM-treated tumors (Figure 5I–L,O). 

### 3.6. Pfkfb3 Genomic Deletion Decreases Tumorigenesis in K-Ras-Driven Lung Tumors In Vivo

**Figure 6:**
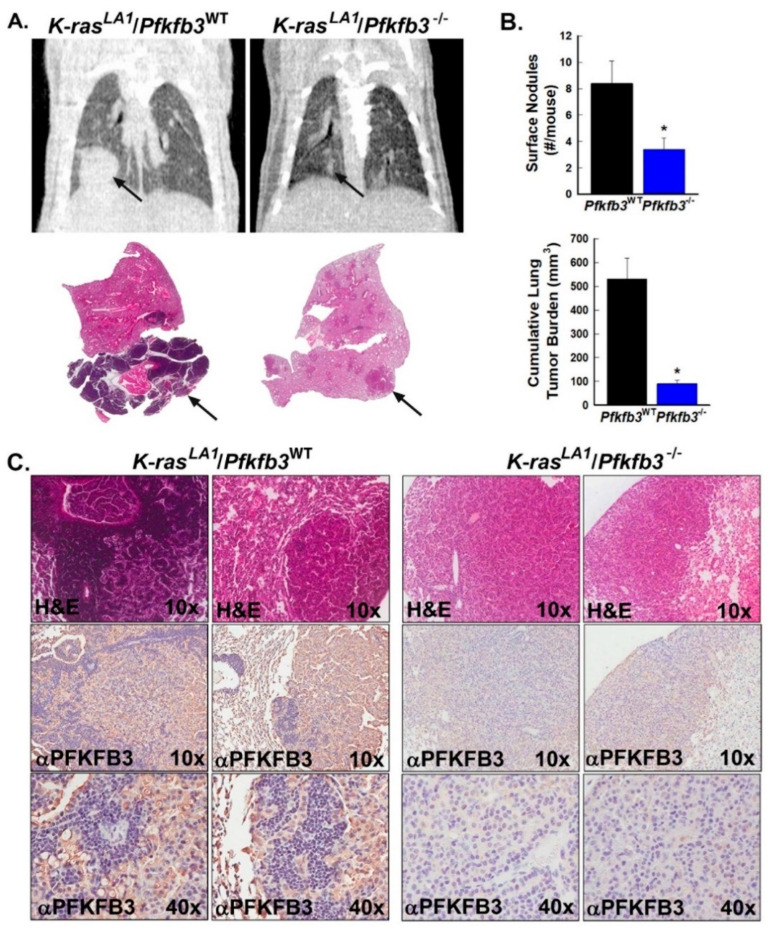
*Pfkfb3* genomic deletion decreases the growth of spontaneous *K-ras*-driven lung tumors in vivo. Groups of *K-ras*^LA1^/Cre/*Pfkfb3*^fl/fl^ mice were treated with corn oil (*K-ras*^LA1^/*Pfkfb3*^WT^) ± TAM (*K-ras*^LA1^/*Pfkfb3*^−/−^) at 6 wks of age (n = 6/group), and mice were followed for clinical signs of distress and tumor growth until the endpoint. At the endpoint, mice were imaged by micro-CT; representative coronal images from *K-ras*^LA1^/*Pfkfb3*^WT^ and *K-ras*^LA1^/*Pfkfb3*^−/−^ mice (upper panels) and lung images (lower panels) are shown, with tumors indicated by black arrows (**A**). Animals were then euthanized, and tumors were enumerated and measured with calipers by gross examination (**B**). Serial sections of tumors from *K-ras^LA1^*/*Pfkfb3*^WT^ and *K-ras^LA1^*/*Pfkfb3*^−/−^ mice were stained with H & E (representative sections, 10× magnification) and examined by immunohistochemistry for PFKFB3 protein expression (representative sections, 10× and 40× magnification) (**C**). Data are expressed as the mean ± SD, * *p* value < 0.05 relative to vehicle controls.

A growing body of evidence indicates that the initiation of neoplastic transformation in tissues requires a shift to glycolysis to support the cellular processes leading to rapid proliferation [42]. To interrogate the role of the PFKFB3 enzyme in the early development and growth of tumors, we elected to evaluate a second model of cancer wherein lung tumors are induced by the somatic activation of a latent oncogenic *K-ras*^G12D^ allele (*K-ras^LA1^* mice) [36] and are highly relevant to human lung cancer due to their oncogenic activation, spontaneous development, and gradual progression. We have observed tumors in *K-ras^LA1^* mice (which have been backcrossed onto a C57BL/6 background) as being first detectable as small surface nodules and adenomas beginning between 10 and 17 weeks of age and growing gradually to substantial volumes by 24–32 weeks of age [43]. In order to evaluate the effects of PFKFB3 loss on tumor initiation and early tumor development, we crossed Cre/*Pfkfb3*^fl/fl^ mice with *K-ras^LA1^* mice and treated *K-ras^LA1^*/Cre/*Pfkfb3*^fl/fl^ mice with vehicle ± TAM at 6 weeks of age for 5 days. Mice were then followed clinically and with micro-CT imaging (Figure 6A). At the endpoint (reached when control animals showed clinical signs of distress including tachypnea and weight loss), we found a lower total lung tumor burden in TAM-treated *K-ras^LA1^*/Cre/*Pfkfb3*^fl/fl^ mice by direct caliper measurements of tumors post-mortem (Figure 6A,B) and also found that these mice showed a reduced number of early lung surface nodules relative to control animals (Figure 6A,B). We examined PFKFB3 expression in tumors and normal lung tissue in TAM-treated and vehicle-treated mice and confirmed decreased PFKFB3 protein expression by immunohistochemistry (Figure 6C). Similar to the *Erbb2* model, to control for unanticipated effects of TAM on tumor growth, we also examined tumor growth in *K-ras^LA1^*/Cre/*Pfkfb3*^+/+^ mice (WT for *Pfkfb3*) administered vehicle ± TAM and found similar growth in both groups (Figure S3).

### 3.7. Pfkfb3 Deletion Decreases Cell Cycle Progression in K-Ras-Driven Lung Tumors

**Figure 7:**
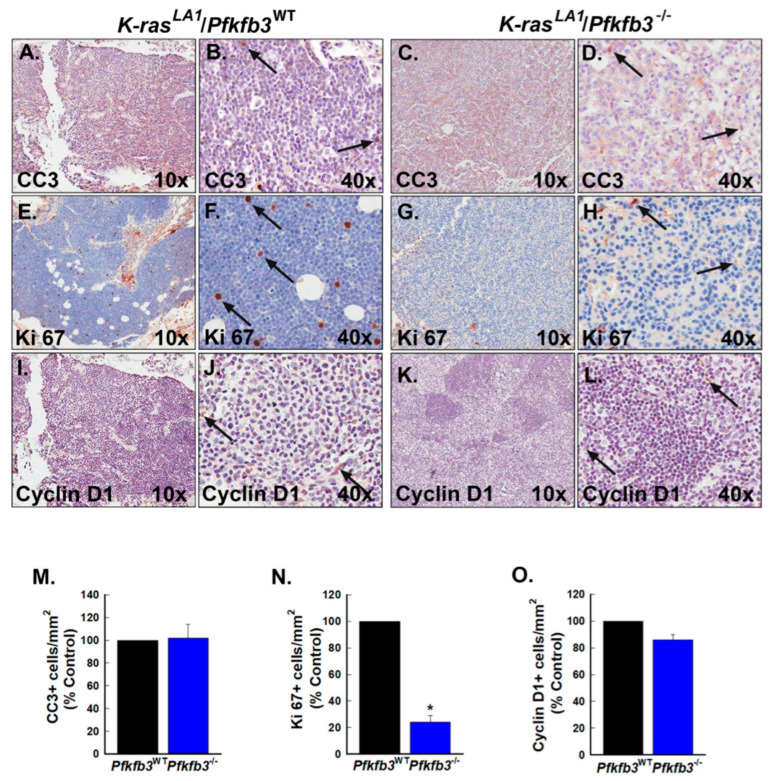
*Pfkfb3* gene deletion causes cell cycle arrest in *K-ras*-driven lung tumors. (**A**–**L**). Serial sections of tumors from *K*-*ras*^LA1^*Pfkfb3*^WT^ and *K*-*ras*^LA1^/*Pfkfb3*^−/−^ mice were examined by immunohistochemistry for CC3, Ki67, and Cyclin D1 expression (representative sections at 10× and 40× magnification shown). Representative positive cells are indicated by black arrows. CC3-, Ki67-, and Cyclin D1-positive cells were enumerated in 5 mm^2^ fields per tumor section from *K*-*ras^LA1^*/*Pfkfb3*^WT^ and *K*-*ras^LA1^*/*Pfkfb3*^−/−^ mice and are presented as indicated (**M**–**O**). Data are expressed as the mean ± SD, * *p* value < 0.05 relative to vehicle.

To determine if the decreased growth of *K-ras*-driven lung tumors in *Pfkfb3-*deleted mice might result from an induction of apoptosis, we first examined serial sections of tumors harvested from vehicle- and TAM-treated mice for CC3 by immunohistochemistry. Similar to our observations in the *Erbb2* model, we found only a minimal increase in the numbers of CC3-positive cells in the TAM-treated lung tumors relative to control tumors (Figure 7A–D,M). To determine whether a decrease in PFKFB3 expression led to an effect on cell cycle progression similar to the *Erbb2* model, we next examined tumors for Ki67-positive cells. We found that the tumors from the TAM-treated mice showed decreased numbers of Ki67-positive cells (Figure 7E–H,N) and additionally showed a decrease in Cyclin D1-positive cells relative to vehicle-treated mice (Figure 7I–L,O) indicating that, similar to the *Erbb2*-driven mammary tumors, PFKFB3 genomic deletion may decrease *K-ras*-driven lung tumor growth by decreasing cell cycle progression. We additionally observed that the differences in CC3 and Cyclin D1 expression were less marked between vehicle- and TAM-treated mice in this model relative to the *Erbb2* model. 

## 4. Discussion

The rapid cell proliferation and growth required for biological processes such as tumor formation and embryogenesis generate a tremendous need for energy and biosynthetic intermediates that is met by a metabolic shift to a high rate of glycolysis [1,44]. Glycolysis additionally plays a key role in multiple other mammalian functions and disease pathologies [24,26,45,46,47,48]. Accordingly, factors that regulate the rate of glycolysis are of critical importance, and their interrogation carries great relevance. The PFKFB family of enzymes has been demonstrated to play a key regulatory role in glucose metabolism due to their production of F26BP, a potent allosteric activator of the rate-limiting PFK-1 enzyme [7,8]. Of the PFKFB isoforms, PFKFB3 is of particular interest due to its high kinase activity which indicates a potentially dominant role for this enzyme in the production of F26BP and therefore in glycolytic regulation. Numerous studies have examined and confirmed the function of the PFKFB3 enzyme in glycolysis in multiple biological processes including an examination of vessel sprouting using a precursor of our current model*,* in xenograft models, and in models with heterozygous expression in vivo [7,13,45,49,50,51,52]. However, the examination of an effective pan-tissue in vivo model of homozygous inducible genomic deletion of *Pfkfb3* is imperative for a complete assessment of the global effects of PFKFB3. The relevance of developing a model has been elevated recently by the development of novel potent inhibitors of PFKFB3 for therapeutic purposes [30,31,53]. Genomic studies to confirm on-target effects and validate the safety of systemic administration of PFKFB3 inhibitors will support the clinical development of these agents. 

To that end, we have now successfully generated a transgenic homozygous floxed *Pfkfb3* mouse line. We have found that these mice exhibit widespread and effective tamoxifen-induced Cre-mediated recombination and genomic deletion of *Pfkfb3* with a resultant marked decrease in PFKFB3 protein expression and PFKFB3-driven F26BP levels in all organs examined. Importantly, the decrease in PFKFB3 expression and activity was not accompanied by adverse effects on life span, organ function, or activity in the mice, corresponding with our previous studies that found no deleterious effects from heterozygous genomic deletion [22] and carrying important implications for the successful development of inhibitors of PFKFB3 for therapeutic applications. We did observe that F26BP was not entirely eliminated in all organs examined, which may be the result of varied organ expression of Cre recombinase as is frequently observed [32] but equally may be the result of the contribution of other co-expressed PFKFB isoforms to intracellular F26BP levels in mammalian tissues [10,34]. 

An important objective of this preliminary interrogation of our model was to validate the efficacy of recombination and deletion of *Pfkfb3* by evaluating the functional effects of decreased PFKFB3. To achieve this goal, we elected to examine tumorigenesis due to the significant reliance of tumor formation and growth on glycolysis [54]. To simulate clinical conditions where a therapeutic intervention would be employed, we first examined the effects of *Pfkfb3* deletion in a model of established oncogene-driven mammary tumor growth. Our results indicated that the resultant decrease in PFKFB3 expression and activity significantly reduced F26BP and growth in established HER2/*Erbb2*+ mammary tumors. We additionally observed decreased glucose uptake by PET scan which we postulate to be the result of reduced PFK-1 activity due to decreased F26BP, which in turn may increase F6P. F6P is in equilibrium with glucose-6-phosphate, an allosteric inhibitor of hexokinase which is required for glucose uptake [55,56,57]. Importantly, these data correspond with previous in vitro work indicating that heterozygous genetic ablation of *Pfkfb3* decreased F26BP and glucose uptake in transformed cells [10]. Further evaluation of TAM-treated tumors post-mortem revealed a significant decrease in indicators of cell cycle progression which correlates closely with observed preclinical in vitro and in vivo effects of targeted PFKFB3 inhibition and therefore serves to confirm the on-target effects of the current PFKFB3 inhibitors in development [30,31]. 

Previous studies from our laboratory determined that oncogene-transformed primary cells required PFKFB3 at wild-type levels of expression to support the development and growth of colonies in soft agar and tumor growth in vivo [10]. These data support the hypothesis that the establishment and growth of transformed cells as a three-dimensional mass in vitro and in vivo may be especially dependent on enhanced glycolytic flux and also indicate a critical role for PFKFB3 in both tumor initiation and growth. Our results examining the effects of homozygous PFKFB3 genomic deletion in the *K-ras^LA1^* model of spontaneous lung tumorigenesis showed that decreased PFKFB3 expression caused a substantial decrease in the formation of early adenomas in addition to suppressive effects on the formation of large tumors. A high rate of glycolysis has been observed in pre-neoplastic tissue both in vitro and clinically, and although still preliminary, our studies indicate that PFKFB3 may play an important role in tumor initiation [42].

We observed that the inhibition of tumor growth caused by *Pfkfb3* deletion was less marked in the *K-ras^LA1^* lung tumor model relative to the *Erbb2* mammary tumor model. Since decreasing PFKFB3 expression and/or activity has previously been demonstrated to effectively suppress proliferation and xenograft growth driven by both of these oncogenes [10,39,58], we speculate that these differences may be attributable to the timing of *Pfkfb3* deletion in these models relative to their tumor onset. Whereas *Pfkfb3* was deleted in *Erbb2* mice after mammary tumors were well established and the period of observation was relatively short due to the rapid growth of the control tumors, *Pfkfb3* was deleted in *K-ras^LA1^* mice at 6 weeks of life and the mice were observed for a prolonged time period. We previously have determined that the PFKFB4 enzyme is co-expressed with PFKFB3 in lung tumors and multiple other tumor types and additionally have observed an increase in PFKFB4 expression when PFKFB3 was silenced [34,59,60]. Based on the presence and high expression of PFKFB4 in these lung tumors and the longer period of decreased PFKFB3 expression, we speculate that PFKFB4 may compensate for the loss of PFKFB3 to support cell cycle progression, proliferation, and tumor growth in this model. Importantly, these data indicate that simultaneous *Pfkfb3* and *Pfkfb4* deletion and/or inhibition may significantly inhibit tumor growth, which is a focus of ongoing studies. 

We acknowledge that our model and our studies have certain limitations. Although our inducible knockout model carries the significant advantage of allowing the homozygous deletion of PFKFB3 expression in adult tissues, which was not possible with our constitutive model [22], it is unable to fully replicate the complexity of human physiology and disease. The results of our experiments must therefore be interpreted with caution when assessing their generalizability to human patients. We also accept that the sample sizes for some of our experiments may be relatively small, which may potentially affect the robustness of our conclusions. In addition, the known variability in timing and organ specificity of Cre recombinase induction [32] may potentially lead to incomplete deletion of *Pfkfb3* in various tissues and deletion at different time points that may have influenced our results. As discussed above, the experimental outcomes also may potentially be affected by compensatory upregulation of other PFKFB isoforms such as PFKFB4 which likely explains the differences that we observed in the growth of the more gradual *K-ras*-driven lung tumor model relative to the *Erbb2* mammary tumor model. Our studies additionally do not address the possibility of alterations in enzymes of the glycolytic pathway and in other tumorigenic pathways due to a decrease in PFKFB3 expression. Last, as the goal of our study was to examine the role of the PFKFB3 enzyme in glycolytic regulation, we did not address the functions of other enzymes and pathways that play important roles in glucose metabolism and tumor growth. These, along with the effects of *Pfkfb3* deletion on their expression and activity, are the subject of ongoing studies.

Taken together, our data thus demonstrate a requirement for PFKFB3-generated F26BP in the initiation and growth of oncogene-driven tumors that are highly relevant to human disease and thereby also confirm the on-target activity of novel PFKFB3 inhibitors. Even more importantly, our studies provide the first characterization of an effective inducible homozygous *Pfkfb3* knockout model that will serve as a powerful tool for understanding and delineating the effects of PFKFB3 regulation in multiple processes that rely on glycolytic metabolism. 

## 5. Conclusions

In summary, these studies detail the successful generation of a novel *Pfkfb3* inducible knockout mouse and the first evaluation of in vivo effects of homozygous pan-tissue *Pfkfb3* gene disruption and also demonstrate that genomic deletion of *Pfkfb3* decreases oncogene-driven tumor growth without adverse systemic effects. These studies establish an effective model for the further examination of the role of PFKFB3 in multiple biological processes and, importantly, validate the further development of novel PFKFB3 inhibitors for therapeutic use. 

## Data Availability

The original contributions presented in the study are included in the article/Supplementary Material; further inquiries may be directed to the corresponding author.

## Supplementary Materials

The following supporting information can be downloaded at https://www.mdpi.com/article/10.3390/cancers16132330/s1: Supplementary Figure S1, which contains original scans of radiographic films from Western blots in Figure 1C; Supplementary Figure S2, which contains data showing tumor growth in *Erbb2*-bearing *Pfkfb3* WT mice; and Supplementary Figure S3, which contains data showing tumor growth in *K-ras*^LA1^-bearing *Pfkfb3* WT mice.
