# Supplementary material for: Genomic Deletion of PFKFB3 Decreases In Vivo Tumorigenesis
Source: Cancers (Basel). 2024 Jun 26;16(13):2330. doi: 10.3390/cancers16132330 (PMC11240529; doi:10.3390/cancers16132330)
Supplement: Supplementary file 1 [file cancers-16-02330-s001.zip › cancers-3063939-supplementary.pdf]

Supplementary Figure S1: Original uncropped Western blot films for Figure 1C.

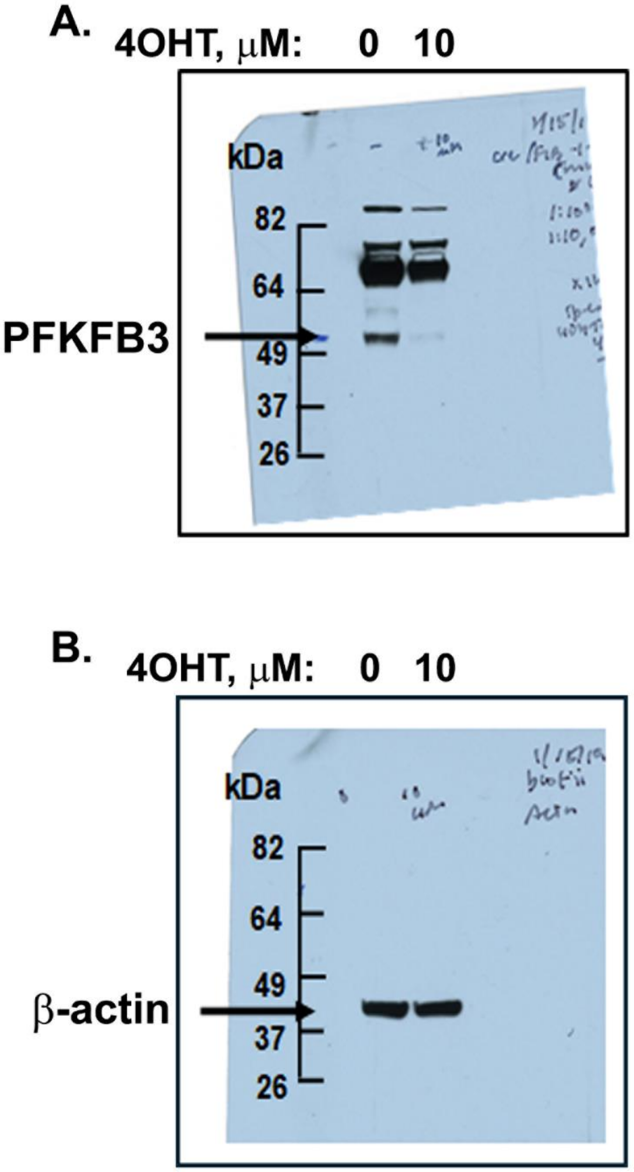

Supplementary Figure S1. Original scans of radiographic films from Western blots in Figure 1.C. Ear fibroblasts isolated from TAM- inducible Cre/$Pfkfb3^{fl/fl}$ mice were plated and exposed to vehicle (ethanol) or 10 μM 4-hydroxytamoxifen (4OHT) for 72 hours then harvested. (**A**). Cellular lysates were examined for PFKFB3 protein expression by Western blot. PFKFB3 is indicated by a black arrow. The visible bands above PFKFB3, importantly, including the strong band at ~ 70 kDa, are non-specific bands that are revealed by the anti-PFKFB3 antibody and are not effectively stripped. (**B**). Cellular lysates were examined for β-actin expression. β-actin is indicated by a black arrow. Of note, β-actin expression was examined first on this membrane therefore the non-specific bands seen in **A.** are not seen in this blot.

Supplementary Figure S2: Tumor growth in *Erbb2*-bearing *Pfkfb3* WT mice is not affected by tamoxifen administration for Cre induction.

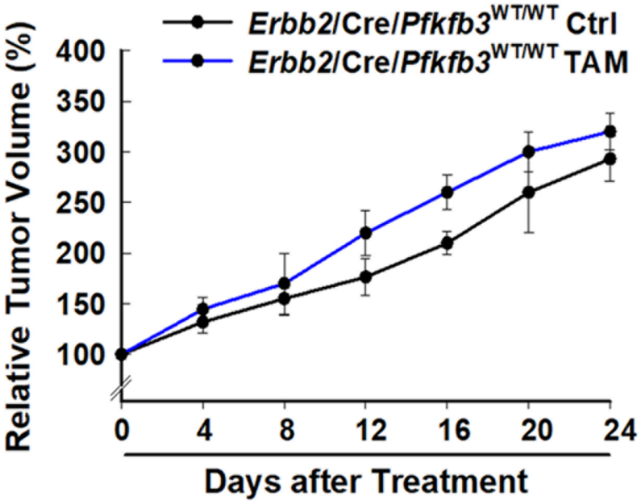

Supplementary Figure S2. Tumor growth in *Erbb2*-bearing *Pfkfb3* WT mice is not affected by tamoxifen administration for Cre induction. Groups of *Erbb2*/Cre/*Pfkfb3*$^{WT/WT}$ mice were followed from appearance of mammary tumors until the tumors were ~100 mg then randomized (n=6/group) to treatment with vehicle (Control, Ctrl) ± TAM. Tumor growth was followed with calipers every 4 days until endpoint (data shown as relative increase in tumor volume from baseline tumor measurements).

Supplementary Figure S3: Tumor growth in *K-ras^{LA1}*-bearing *Pfkfb3* WT mice is not affected by tamoxifen administration for Cre induction.

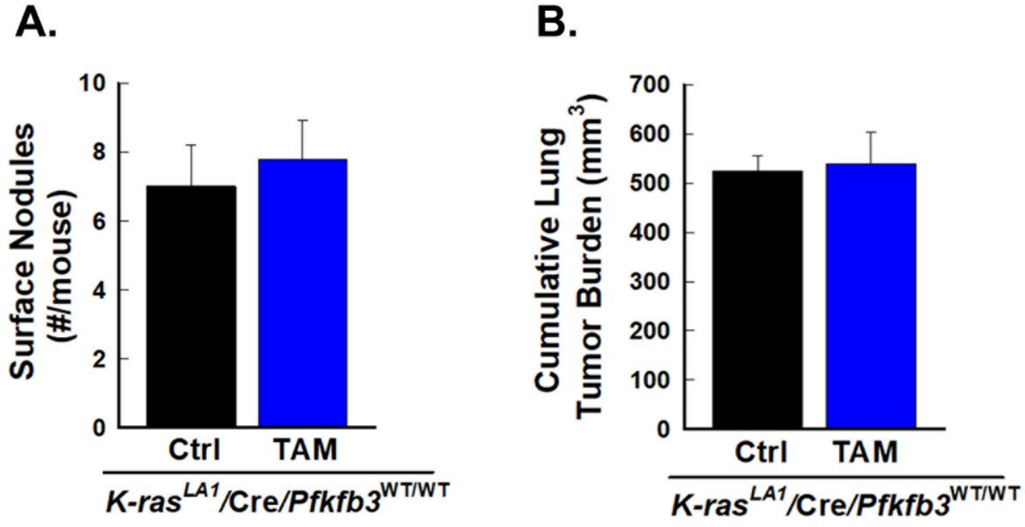

Supplementary Figure S3. Tumor growth in *K-ras^{LA1}*-bearing *Pfkfb3* WT mice is not affected by tamoxifen administration for Cre induction. Groups of *K-ras^{LA1}*/Cre/*Pfkfb3*$^{WT/WT}$ mice were treated with corn oil (Control, Ctrl) ± TAM at 6 wks of age (n=6/group) and were followed for clinical signs of distress and tumor growth until endpoint. At endpoint, tumors were enumerated (**A**) and measured with calipers by gross examination (**B**).
